# Low-temperature atomic layer epitaxy of AlN ultrathin films by layer-by-layer, *in-situ* atomic layer annealing

**DOI:** 10.1038/srep39717

**Published:** 2017-01-03

**Authors:** Huan-Yu Shih, Wei-Hao Lee, Wei-Chung Kao, Yung-Chuan Chuang, Ray-Ming Lin, Hsin-Chih Lin, Makoto Shiojiri, Miin-Jang Chen

**Affiliations:** 1 Department of Materials Science and Engineering, National Taiwan University, Taipei, Taiwan, R.O.C; 2 Department of Electronic Engineering, Chang Gung University, Tao-Yuan 333, Taiwan, R.O.C; 3 Department of Radiation Oncology, Chang Gung Memorial Hospital, Tao-Yuan 333, Taiwan, R.O.C; 4 Kyoto Institute of Technology, Kyoto, Japan

## Abstract

Low-temperature epitaxial growth of AlN ultrathin films was realized by atomic layer deposition (ALD) together with the *layer-by-layer, in-situ* atomic layer annealing (ALA), instead of a high growth temperature which is needed in conventional epitaxial growth techniques. By applying the ALA with the Ar plasma treatment in each ALD cycle, the AlN thin film was converted dramatically from the amorphous phase to a single-crystalline epitaxial layer, at a low deposition temperature of 300 °C. The energy transferred from plasma not only provides the crystallization energy but also enhances the migration of adatoms and the removal of ligands, which significantly improve the crystallinity of the epitaxial layer. The X-ray diffraction reveals that the full width at half-maximum of the AlN (0002) rocking curve is only 144 arcsec in the AlN ultrathin epilayer with a thickness of only a few tens of nm. The high-resolution transmission electron microscopy also indicates the high-quality single-crystal hexagonal phase of the AlN epitaxial layer on the sapphire substrate. The result opens a window for further extension of the ALD applications from amorphous thin films to the high-quality low-temperature atomic layer epitaxy, which can be exploited in a variety of fields and applications in the near future.

Atomic layer deposition (ALD) is an emerging and attractive technique for preparing nanoscale ultrathin films because of high uniformity over a large area, accurate thickness control, low defect density, excellent conformality and step coverage, *etc*., due to the self-limiting and layer-by-layer growth^1^. Thus ALD has been widely applied in a variety of nanoscale devices such as transistors^2^, memories^3^, medical devices^4^, fuel cells^5^,^6^, and solar cells^7^. However, it is necessary to keep a low deposition temperature for self-limiting ALD growth in order to prevent the thermal decomposition or desorption of precursors^8^. Hence the as-deposited thin films prepared by ALD are generally of amorphous-like structure^9^. As compared with amorphous films, crystalline films (or even single-crystalline epitaxial layers) are more preferable in terms of significantly improved material properties, especially the optical and electrical characteristics. Therefore, it is desirable to increase the crystallinity of ultrathin films prepared by ALD. In order to improve the crystallinity of thin films or achieve an epitaxial layer, the atoms in the films need to get sufficient energy for moving or migrating to bind with the nearest neighbors, and so conventional epitaxial growth techniques are usually carried out at a high temperature. However, the high deposition temperature in the ALD process gives rise to desorption of surface species, or precursor decomposition (as resulting in the chemical vapor deposition (CVD) mode). Therefore, instead of high deposition temperature, another way to provide the energy for adatom migration is needed for ALD to achieve high-quality crystalline films. The plasma treatment during deposition is a feasible method where the desired crystallization energy can be transferred to the deposited film from the incident ions or radicals^10^. Actually, the substrate temperature rise caused by the radio-frequency discharge plasma has been reported^11^. Another example of the ion bombardment during deposition is the decrease in the epitaxial temperature by 120 °C for Si grown by molecular beam epitaxy when the low-energy ions were supplied^12^.

Since ALD is a layer-by-layer process, the *in-situ* plasma treatment can be applied in each ALD cycle. Hence, the surface temperature of the sample might increase as a result of the plasma treatment, leading to the annealing effect during the layer-by-layer ALD growth. Accordingly, the *in-situ,* layer-by-layer plasma treatment in each ALD cycle is proposed in this paper and denoted terminologically as “atomic layer annealing” (ALA). The ALA treatment may enhance the adatom movement and migration on the surface, which is favorable for the improved crystallization of the deposited films. Another effect caused by ALA is to facilitate the removal of the ligands of the chemisorbed precursors. Consequently, the physical or chemical properties of film surface can be tailored by the ALA treatment, leading to the promotion of crystallization of the films at relatively low deposition temperatures.

Aluminum nitride (AlN) has attracted a lot of attention due to its distinguished material characteristics including wide direct band gap, high piezoelectric response, and good stability at high temperature. Hence AlN has been widely applied in ultraviolet light-emitting diodes^13^, resonators^14^, and sensors^15^. For example, the two-dimensional electron gas (2DEG) near the Al(Ga)N/GaN interface has been recently exploited in high-electron-mobility transistors (HEMTs)^16^. In general, high-quality AlN epilayers were conventionally grown by metal-organic chemical vapor deposition (MOCVD). However, a very high temperature (>900 °C) is required to grow high-quality AlN epilayers using MOVCD. Besides, a large thickness up to ~600 nm is needed to achieve a fully coalesced, continuous MOCVD AlN hetero-epitaxial layer on the sapphire substrate^17^,^18^. The high growth temperature and fully coalesced thickness result in practical limitations on applications of MOCVD AlN epilayers.

Because of the distinguished advantages of ALD, a lower deposition temperature and a smaller coalesced thickness could be expected in the AlN thin films prepared by ALD. Thus the deposition and characteristics of the AlN thin films prepared by ALD have been intensively investigated recently^19^,^20^,^21^,^22^,^23^,^24^. However, owing to the low deposition temperature, the ALD-deposited AlN thin films are generally amorphous or polycrystalline, resulting in a low polar field which deteriorates the performance of HEMTs. Therefore, the improvement of crystallinity of the AlN layer prepared by ALD is highly desirable.

In this study, we make a proposal of a novel technique and concept of ALA in the ALD process to achieve high-quality epitaxial growth of AlN at a low deposition temperature of 300 °C. By introducing an *in-situ*, layer-by-layer Ar plasma treatment into each ALD cycle, the crystallinity of ALD-deposited AlN films was improved significantly, and a high-quality AlN epilayer with a thickness of only ~30 nm was achieved. The mechanism of ALA was also experimentally investigated and discussed, indicating that the crystallization takes place due to the surface heating caused by the *in-situ* ALA. Finally, the high-quality AlN layer prepared with the ALA treatment was grown on a bulk GaN epilayer, revealing the 2DEG characteristics near the AlN/GaN heterojunction with the significant improvement of mobility and sheet electron concentration by the *in-situ* ALA.

## Result and Discussion

### Atomic layer annealing (ALA)

**Figure 1:**
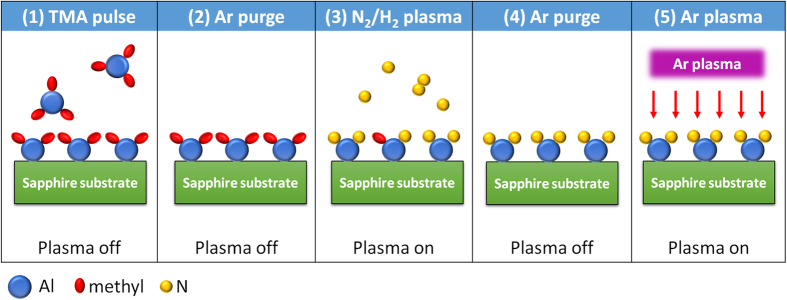
The schematic diagram of the modified ALD cycles for atomic layer epitaxy, with an additional step (5) of the Ar plasma treatment for *in-situ* ALA.

The scheme of the ALD cycles for growing the AlN epilayers using the *in-situ* ALA is plotted in Fig. 1. The standard ALD cycle consisted of the following steps: (1) TMA pulse, (2) Ar purge, (3) remote N_2_/H_2_ plasma, and (4) Ar purge. As for the ALD cycle including the *in-situ* ALA, an additional step (5) of the Ar plasma treatment was added after the step (4) in each ALD cycle. In this way, each as-deposited monolayer formed by the standard ALD cycle (1)-(4) was modulated by the subsequent Ar plasma treatment (5). Hereafter the “standard” AlN layer is an AlN layer that was prepared without the additional step (5) for the ALA treatment.

### The growth rate, uniformity, roughness, and surface morphology of the ALA-treated AlN layer

Figure S1 in the Supplementary Information shows the thickness of the ALA-treated AlN layer as a function of the applied ALD cycles, revealing a linear dependence between the film thickness and the applied ALD cycles including the additional step (5) of the Ar plasma treatment. The result indicates that the thickness of films treated with *in-situ* ALA can be precisely and digitally controlled by the number of the applied ALD cycles. The growth rate of the ALA-treated AlN layer deduced from Figure S1 was ~0.9 Å per ALD cycle. Figure S2 in the Supplementary Information illustrates the uniformity of the film thickness of the ALA-treated AlN layer on the sapphire substrate with an effective area corresponding to a 6” diameter. The percent non-uniformity of the film thickness is only ±1.1%, indicating high uniformity of the AlN layer prepared with the ALA treatment. The surface roughness of the standard AlN and ALA-treated AlN layers characterized by AFM is shown in Figure S3 in the Supplementary Information. The root mean square (RMS) of the surface roughness of the ALA-treated AlN layer is 0.663 nm, which is slightly higher than that (0.223 nm) of the standard AlN layer but still in the same order of magnitude. The increase of surface roughness could be explained by the enhancement of grain size in the AlN layer treated with *in-situ* ALA as a result of the improvement of crystallinity, which is mentioned in the following section. The SEM image of the ALA-treated AlN layer is shown in Figure S4 in the Supplementary Information. It can be observed that the surface of the ALA-treated AlN layer is very smooth without any obvious pits or pinholes, which might be attributed to the layer-by-layer growth of ALD. In addition, the high conformality of the films prepared by ALD results in the filling of surface pits and pinholes by the following deposited layers, which is beneficial to reducing the density of pits or pinholes on the surface. Furthermore, the thermal stress induced by the difference in the thermal expansion coefficient between AlN and sapphire may lead to the formation of pits and cracks^25^. Since only a few layers of atoms near the surface were heated in the case of *in-situ* ALA, the pits and cracks caused by the thermal stress were much suppressed. Therefore, the ALA-treated AlN layer exhibited a very smooth and uniform surface which is nearly free of pits and cracks.

### Crystallinity of the ALA-treated AlN layer

**Figure 2:**
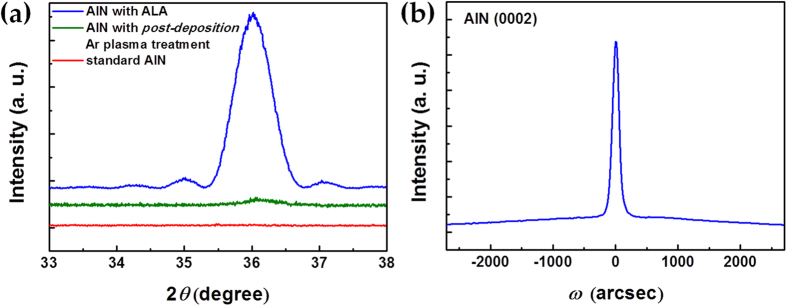
(**a**) XRD *θ-*2*θ* scans of the standard AlN layer, the AlN layer treated with *post-deposition* Ar plasma, and the ALA-treated AlN layer. (**b**) XRD *ω*-scan rocking curve of the (0002) AlN peak of the ALA-treated AlN layer with a thickness of only ~30 nm.

The *θ-*2*θ* XRD patterns of the standard AlN and ALA-treated AlN layers are shown in Fig. 2(a). In order to clarify the effect of ALA, the XRD pattern of the AlN layer prepared with the *post-deposition* Ar plasma treatment was also shown in Fig. 2(a) for comparison. The plasma power and total treatment time of the *post-deposition* Ar plasma treatment (300 W, 4000 sec) were controlled to be identical to those of ALA. Significant reflection at ~36.0° associated with the (0002) hexagonal AlN in Fig. 2(a) indicates that the ALA-treated AlN layer was well-crystallized with a wurtzite structure. As compared with the standard AlN layer and the AlN layer prepared with the *post-deposition* Ar plasma treatment, the remarkable enhancement of the XRD peak intensity indicates that the ALA treatment greatly improves the crystallinity of the AlN ultrathin film. Notice that multiple satellite peaks were also clearly observed around the (0002) main peak of the ALA-treated AlN layer. These satellite peaks originates from the interference due to an ultrathin hetero-epitaxial layer^26^, which provides further evidence of high crystallinity and smooth surface of the ALA-treated AlN layer. The significant difference in the crystallinity between the AlN layers prepared with the ALA and the *post-deposition* Ar plasma treatments can be attributed to the enhanced adatom migration on the surface by the layer-by-layer, *in-situ* ALA treatment. During the ALA treatment in each ALD cycle, the loosely bound adatoms on the surface are prone to migrate, leading to high crystallinity of the ALA-treated AlN layer. Figure 2(b) shows the *ω*-scan rocking curve of the ALA-treated AlN layer with a thickness of only ~30 nm. The full width at half-maximum (FWHM) of XRD *ω*-scan rocking curves is only 144 arcsec, which is close to the typical value of a thick (~1 μm) AlN epilayer grown by MOCVD at a high temperature of 900~1100 °C^27^. Hence, Fig. 2 clearly reveals that the crystallinity of AlN is improved dramatically by the ALA treatment, and high-quality AlN epilayers with a thickness of a few tens of nm can be achieved at a low growth temperature as low as 300 °C.

**Figure 3:**
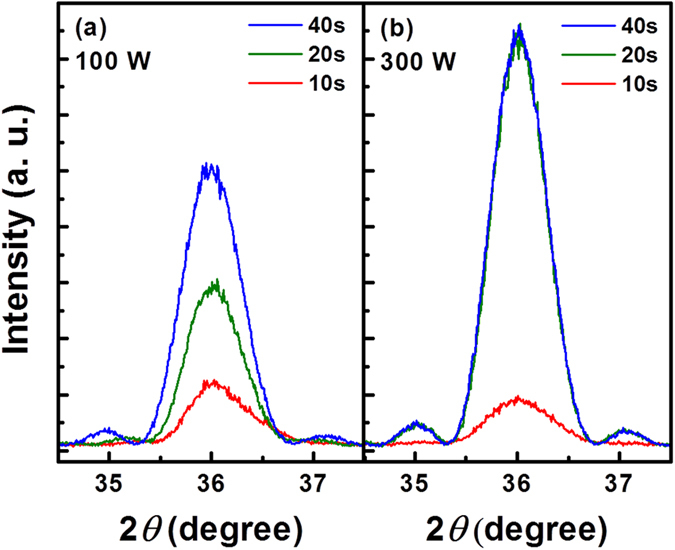
The *θ-*2*θ* XRD patterns of the AlN layers treated with the Ar plasma power of (**a**) 100 W, and (**b**) 300 W, and the treatment time of 10, 20, and 40 sec, during the *in-situ* ALA in each ALD cycle.

Figure 3 shows the *θ-*2*θ* XRD patterns of the AlN layers treated with different Ar plasma power and time during the *in-situ* ALA in each ALD cycle. When the Ar plasma power was kept at 100 W (Fig. 3(a)), the XRD peak intensity increased with the plasma treatment time from 10 sec to 40 sec, indicating the improvement in crystallinity of the AlN layer with the increase of the plasma treatment time. As the Ar plasma power was raised to 300 W (Fig. 3(b)), the XRD peak intensity increased significantly with the increment of the plasma treatment time from 10 sec to 20 sec, and then saturated when the plasma treatment time was greater than 20 sec. Notice that the FWHM of all the (0002) AlN XRD peaks shown in Fig. 3(a,b) is almost the same. Since the vertical crystallite size is limited by the thickness of the film with high crystallinity, the approximately identical thickness of the AlN layers leads to indistinguishable difference between the FWHM of the (0002) AlN XRD peaks. As for the saturation of the crystallinity of the AlN layer when the plasma treatment time was greater than 20 sec (Fig. 3(b)), it can be understood that the thermal crystallization would reach saturation as the annealing time is sufficient^28^. The result shown in Fig. 3 indicates that the increase in the dose of plasma treatment, including the plasma power and time, is capable of enhancing the effect of *in-situ* ALA.

**Figure 4:**
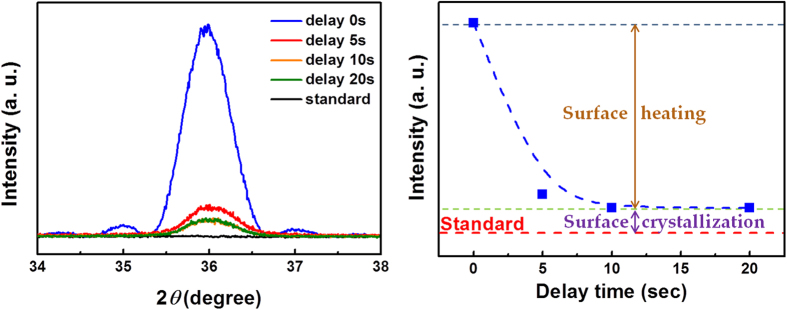
(**a**) The *θ-*2*θ* XRD pattern of the ALA-treated AlN layers with the delay time of 0, 5, 10, and 20 sec. (**b**) The AlN (0002) XRD peak intensity as a function of the delay time between the Ar plasma treatment and the following TMA pulse.

In order to confirm the effect of *in-situ* ALA, the duration between the Ar plasma treatment (5) and the following TMA pulse (1) (see Fig. 1), which is defined as the delay time, was changed from 0 to 20 sec in each ALD cycle. Figure 4(a) shows the *θ-*2*θ* XRD patterns of the ALA-treated (300 W, 20 s) AlN layers with different delay times. The AlN (0002) XRD peak intensity as a function of the delay time is plotted in Fig. 4(b). It is seen that the XRD peak intensity decreases significantly with the delay time, but is still higher than that of the standard AlN layer without the ALA treatment as the delay time is 20 sec. Hence it can be deduced that two mechanisms take place for the crystallinity improvement during the ALA treatment: one depends on the delay time and the other is independent of the delay time. During the *in-situ* ALA, the AlN layer gets the energy from the incident Ar ions and radicals, which promotes the migration of adatoms on the surface so as to bind with the atoms on the underlayer and the nearest neighboring adatoms. Thus the crystallization occurs in the deposited atomic layer during the Ar plasma treatment. This process termed as “surface crystallization” is supposed to be independent to the delay time. On the other hand, the energy transferred from the Ar plasma effectively results in the increase of the surface temperature. This “surface heating” effect will enhance the reactivity of the following TMA precursors adsorbed on the surface, leading to the higher effectiveness for ligands desorption from the precursors as well as the adatom migration^29^. Thus the effect of surface heating on the following adsorbed TMA precursor strongly depends on the delay time, and eventually decays with the increase of the delay time as a result of heat dissipation into the substrate and the surroundings. Therefore, the XRD peak intensity decays quickly with the delay time. Because the surface crystallization takes place during the Ar plasma treatment, the crystallinity of the ALA-treated AlN layer is still higher than that of the standard AlN layer without the ALA treatment, even with a very long delay time of 20 sec between the Ar plasma treatment and the following TMA pulse.

**Figure 5:**
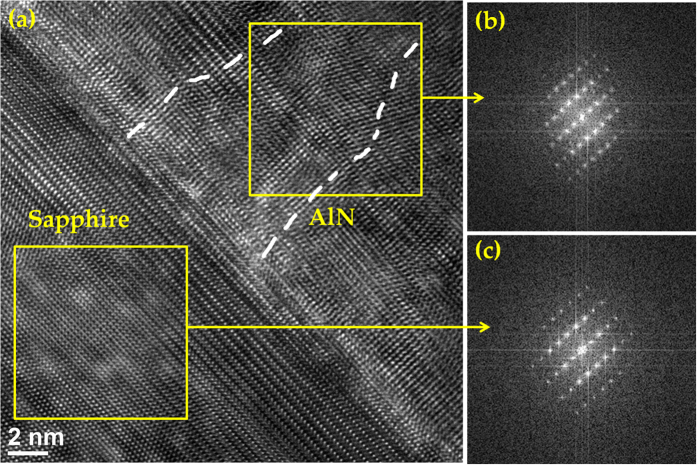
(**a**) HRTEM image of the ALA-treated AlN epilayer grown on the sapphire substrate. (**b,c**) The FFT diffractograms of the areas enclosed in the AlN layer and sapphire, respectively. The threading dislocations are indicated by the dotted lines in (**a**).

HRTEM was performed on the ALA-treated AlN layer to further investigate the microstructure of the film and the characteristics of the interface. Figure 5(a) shows the cross-sectional HRTEM image at the AlN/sapphire interface, revealing a sharp interface and a well-defined crystal lattice of AlN. The fast Fourier transform (FFT) diffractograms shown in Fig. 5(b,c) refer to the areas enclosed in the region of the AlN layer and the sapphire substrate, respectively. The diffractogram in Fig. 5(b) reveals a high-quality single crystal of hexagonal AlN with [0001] prefer orientation perpendicular to the substrate surface. Comparing the FFT patterns of the AlN layer and sapphire substrate indicates the epitaxial relationship of AlN with respect to the substrate: [0001]_AlN_//[0001] _sapphire_ and [1
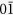
0]_AlN_//[11
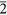
0]_sapphire_. Hence the growth of the ALA-treated AlN layer proceeds epitaxially layer-by-layer on the underlying surface from just the first layer on the substrate. Because of the lattice mismatch, the misfit dislocations can be formed at the interface between the sapphire substrate and the AlN epitaxial layer, and then extend as threading dislocations into the upper layer. The disarray of the AlN lattice fringes as indicated by the dotted lines in Fig. 5(a) may be ascribed to threading dislocations.

### Chemical composition and depth profile analysis of the ALA-treated AlN layer

**Figure 6:**
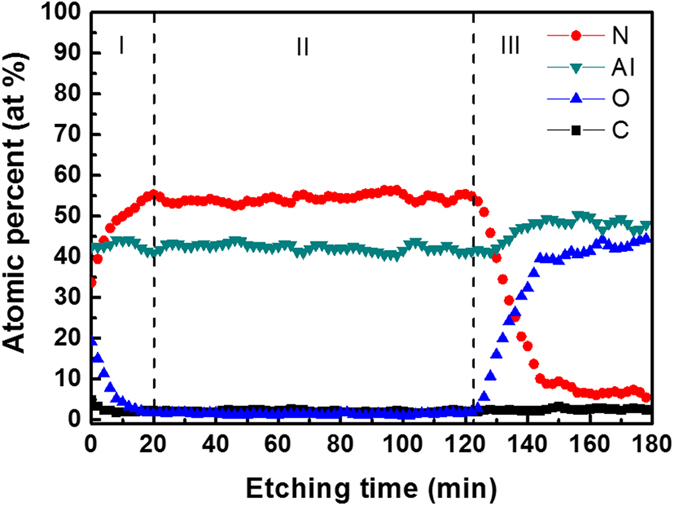
The Auger depth profile of the ALA-treated AlN layer.

The Auger survey spectrum of the ALA-treated AlN layer (as show in Figure S5 in the Supplementary Information) exhibited four elements: carbon, nitrogen, oxygen, and aluminum. Figure 6 shows the Auger depth profile of each chemical composition in the ALA-treated AlN layer. Signals of contamination such as carbon and oxygen in the region I (as indicated in Fig. 6) decrease sharply from the surface and approach to the detection limit of AES when the etching time is greater than 1 min, indicating that the carbon and oxygen in the ALA-treated AlN layer are mainly ascribed to surface contamination and oxidation caused by the ambient atmosphere after the film deposition. In the region II, the nitrogen and aluminum signals remain almost identical down to the interface between the AlN layer and the sapphire substrate (region III), suggesting that the composition of AlN is quite uniform across the layer. The slightly higher nitrogen content than aluminum (Al:N~1:1.2) in the region II might result from the plasma enhanced dissociation of nitrogen during the AlN deposition^30^,^31^.

### Refractive index of the ALA-treated AlN layer

**Figure 7:**
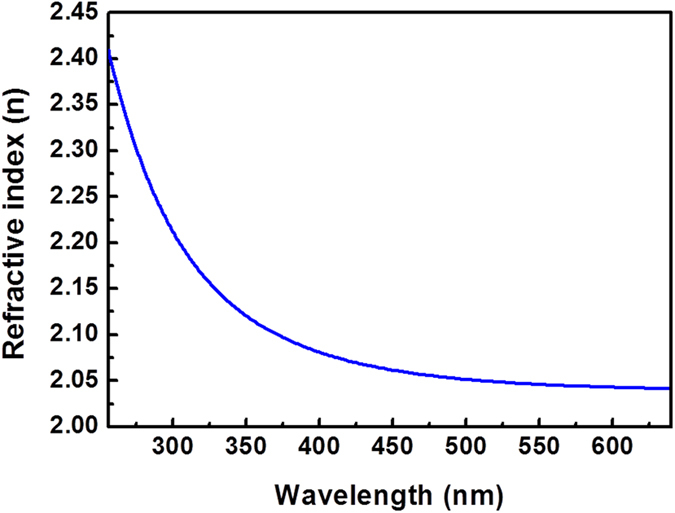
The refractive index as a function of wavelength of the ALA-treated AlN film.

The refractive index of the ALA-treated AlN layer was extracted from the spectroscopic ellipsometer (SE). Figure 7 shows the dispersion of the refractive index of the ALA-treated AlN layer in the wavelength range between 256 and 640 nm. The SE data was fitted by the quantum mechanical model, which is widely used for semiconductor materials. The refractive index of the ALA-treated AlN layer at λ = 633 nm is 2.04 and the bandgap energy given by the quantum mechanical model is 6.01 eV, which is close to the reported values of high-quality epitaxial AlN films^32^,^33^.

### Electrical properties of the ALA-treated AlN layer

**Figure 8:**
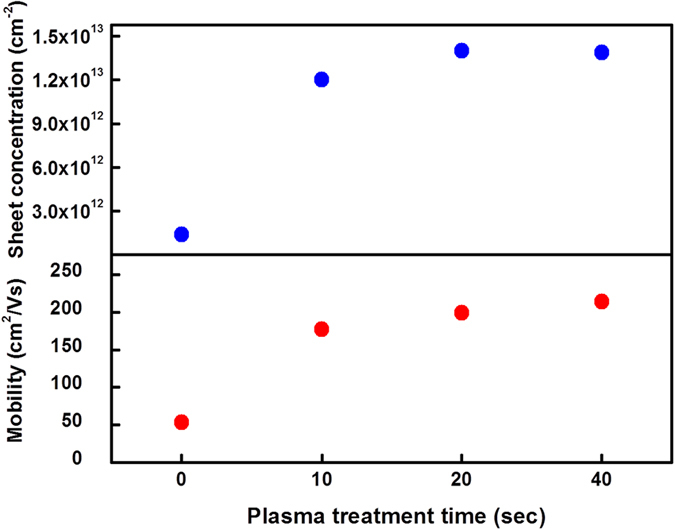
Sheet electron concentration and mobility of the AlN/GaN heterojunction, where the AlN layer was treated with the *in-situ* ALA with different plasma time of 0, 10, 20, and 40 sec.

In order to investigate the electrical properties of the AlN ultrathin films prepared with the ALA treatment, 10 nm-thick layers of standard AlN and ALA-treated AlN (300 W) with different plasma treatment time were deposited on 4 μm-thick undoped-GaN epilayers which were grown by MOCVD. Figure 8 shows the sheet electron concentration (*n*_*s*_) and mobility of the AlN/GaN heterojunction. As compared with the standard AlN/GaN heterojunction with *n*_*s*_ of only 1.6 × 10^12^ cm^−2^, the *n*_*s*_was greatly increased by one order of magnitude to 1.4 × 10^13^ cm^−2^ in the ALA-treated AlN/GaN heterojunction as the plasma treatment time was greater than 10 sec. The mobility also increased from 53.3 to 214.1 cm^2^V^−1^s^−1^ with the ALA treatment on the AlN layer. The remarkable enhancement of the AlN crystallinity by the ALA treatment is responsible for the significant increase of the *n*_*s*_ and mobility. The high crystalline quality of the AlN layer will induce the spontaneous polarization and strain-induced piezoelectric field^34^. Hence the enhancement of AlN (0002) preferred orientation by the ALA treatment improves the polarization field in the [0001] direction, leading to the increment of the *n*_*s*_ and mobility. Therefore the significant increase of the sheet electron concentration and mobility could be attributed to the onset of the 2DEG near the AlN/GaN heterojunction prepared with the ALA treatment. Note that the increase of the *n*_*s*_ and mobility saturates when the plasma treatment time is greater than 20 sec, which is in good agreement with the saturation of the AlN crystallinity as shown in Fig. 3(b).

## Conclusion

In summary, a novel concept and approach termed “atomic layer annealing” (ALA) was proposed to realize the low-temperature atomic layer epitaxy of ultrathin films. ALA is the layer-by-layer, *in-situ* Ar plasma treatment applied in each ALD cycle to significantly improve the crystal quality of AlN ultrathin films, converting the structure from the amorphous phase to single-crystalline epitaxial layer. This is attributed to the surface crystallization caused by moving or migration of adatoms on the surface, so as to bind with the atoms on the underlayer and the nearest neighboring adatoms as the crystal. In addition, the surface heating effect that enhances the reactivity of the following precursors also occurs for the improvement of film crystallinity during the ALA treatment. Thus a high-quality AlN epilayer with a thickness of a few tens of nm was achieved by the ALA at a low deposition temperature of 300 °C. The XRD and HRTEM characterization clearly revealed a high-quality single-crystal hexagonal AlN epilayer. The 2DEG characteristics of the AlN/GaN heterojubction was also accomplished by the ALA treatment on the AlN layer. The result demonstrates that the layer-by-layer, *in-situ* ALA in each ALD cycle is a promising technique to achieve high-quality atomic layer epitaxy of nanoscale ultrathin films at a low temperature, which extends the applications of ALD tools from the conventional thin film deposition to high-quality epitaxial growth.

## Method

### ALD process

The AlN epilayers were grown on (0001) sapphire substrates by plasma-enhanced ALD. Trimethylaluminum (TMA) and remote N_2_/H_2_ plasma were utilized as the precursors for aluminum and nitrogen, respectively, which were delivered into the reaction chamber by the Ar carrier gas. The deposition was conducted at a low temperature of 300 °C.

### Measurement

The thickness and refractive index of the AlN layers were measured by the spectroscopic ellipsometer (SE, Elli-SE, Ellipso Technology). The crystalline phase and quality of the AlN thin films were characterized with the high-resolution transmission electron microscopy (HRTEM, FEI Tecnai G2 F20) and X-ray diffraction (XRD, PANalytical X’Pert Pro and Bruker D8) in *θ-*2*θ* and *ω*-scan (rocking curve) modes. Atomic force microscopy (AFM, NT-MDT) was used to evaluate the surface roughness of the AlN layers. The chemical composition and depth profile of the ALA-treated AlN layer were measured by the Auger electron spectroscopy (AES, JAMP-9510F Field Emission Auger Microprobe, JEOL). The acceleration voltage, electron current, and incident angle were 5 kV, 4 nA, and 60°, respectively. The sample was etched by 3 keV Ar ions and the duration of each etching cycle in the AES measurement was 3 sec. Scanning electron microscope (SEM, FEI Helios Nanolab 600i dual beam system) was used to examine the surface morphology of the ALA-treated AlN layer. The electrical properties of 2DEG were measured by the Ecopia HMS-3000 Hall-effect measurement system in the van der Pauw configuration at room temperature.

## Additional Information

**How to cite this article**: Shih, H.-Y. *et al*. Low-temperature atomic layer epitaxy of AlN ultrathin films by layer-by-layer, *in-situ* atomic layer annealing. *Sci. Rep.* **7**, 39717; doi: 10.1038/srep39717 (2017).

**Publisher's note:** Springer Nature remains neutral with regard to jurisdictional claims in published maps and institutional affiliations.

## Supplementary Material

Supplementary Information
