# Supplementary material for: Low-temperature atomic layer epitaxy of AlN ultrathin films by layer-by-layer, in-situ atomic layer annealing
Source: Sci Rep. 2017 Jan 3;7:39717. doi: 10.1038/srep39717 (PMC5206640; doi:10.1038/srep39717)
Supplement: Supplementary Information [file srep39717-s1.doc]

**Low-temperature atomic layer epitaxy of AlN ultrathin films by layer-by-layer, *in~~-~~situ* atomic layer annealing**

**Supplementary Information**

*Huan-Yu Shih^a^, Wei-Hao Lee^a^, Wei-Chung Kao^a^, Yung-Chuan Chuang^a^,*

*Ray-Ming Lin^b,c^, Hsin-Chih Lin^a^, Makoto Shiojiri^d^, and Miin-Jang Chen^a^,**

^a^ Department of Materials Science and Engineering, National Taiwan University, Taipei, Taiwan

^b^ Department of Electronic Engineering, Chang Gung University, Tao-Yuan 333, Taiwan, R.O.C.

^c^ Department of Radiation Oncology, Chang Gung Memorial Hospital, Tao-Yuan 333, Taiwan, R.O.C.

^d^ Kyoto Institute of Technology, Kyoto, Japan.

*^*^* Authors to whom any correspondence should be addressed.

E-mail: mjchen@ntu.edu.tw

Phone: +886-2-233665301

**
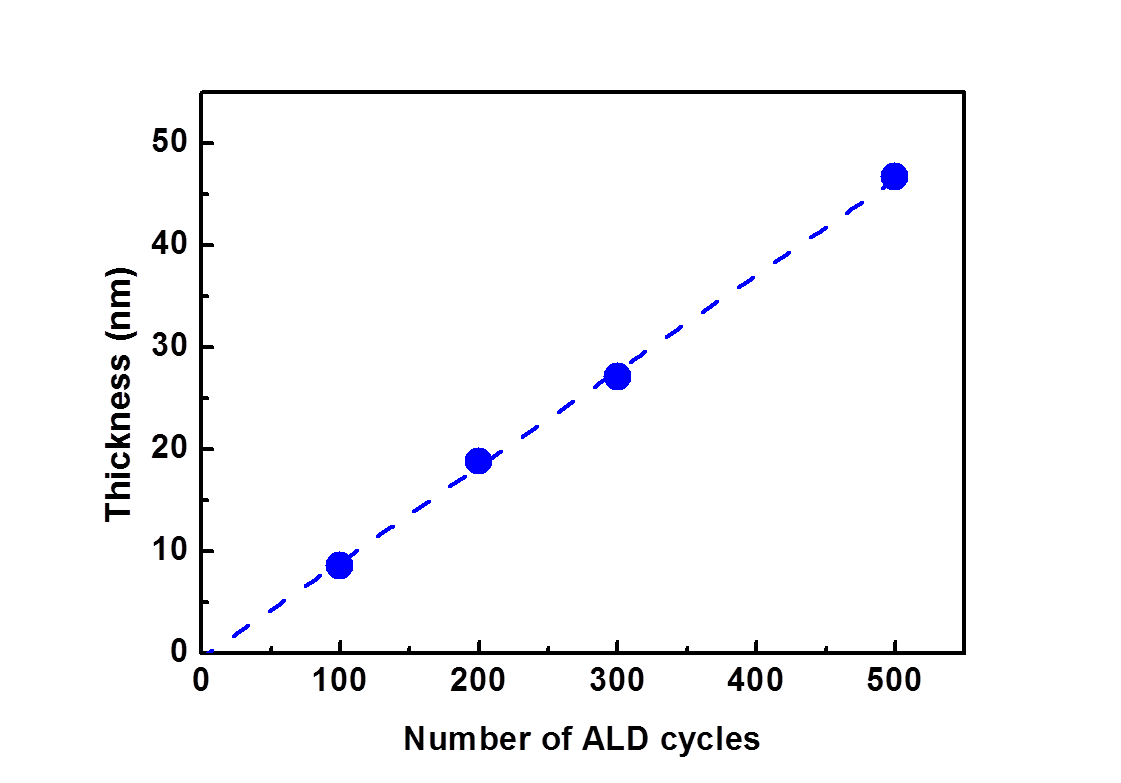
**

**Figure S1.** The thickness of the ALA-treated AlN layer as a function of applied ALD cycles.


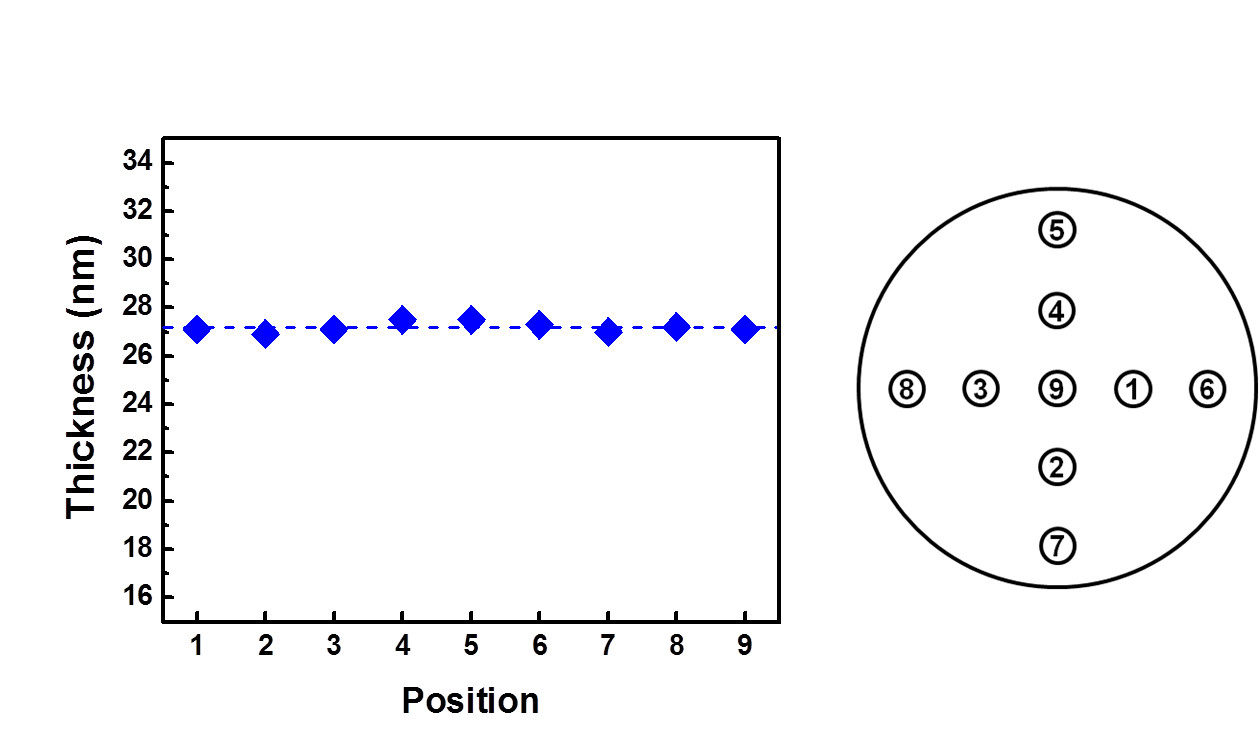


**Figure S2.** The thickness of the ALA-treated AlN layer at different positions on the sapphire substrate with an effective area of 6-inch diameter.


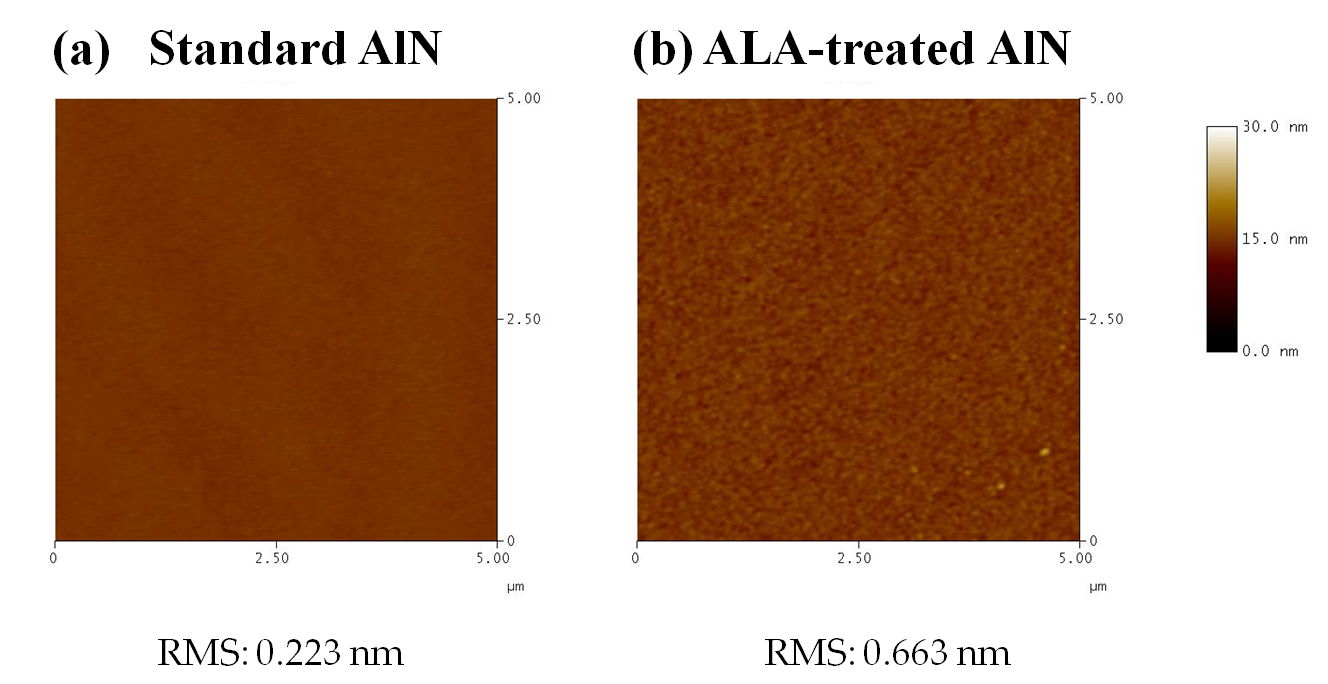


**Figure S3.** AFM images of the (a) standard AlN and (b) ALA-treated AlN layers.


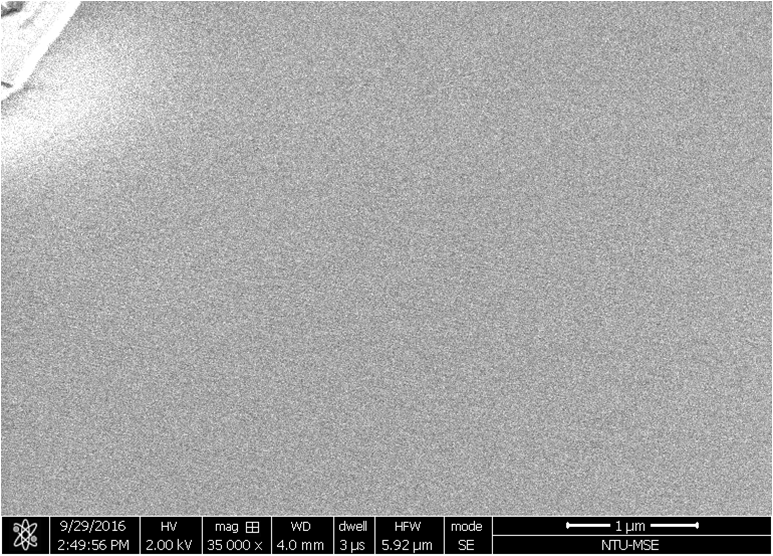


**Figure S4** SEM micrograph of the ALA-treated AlN layer.


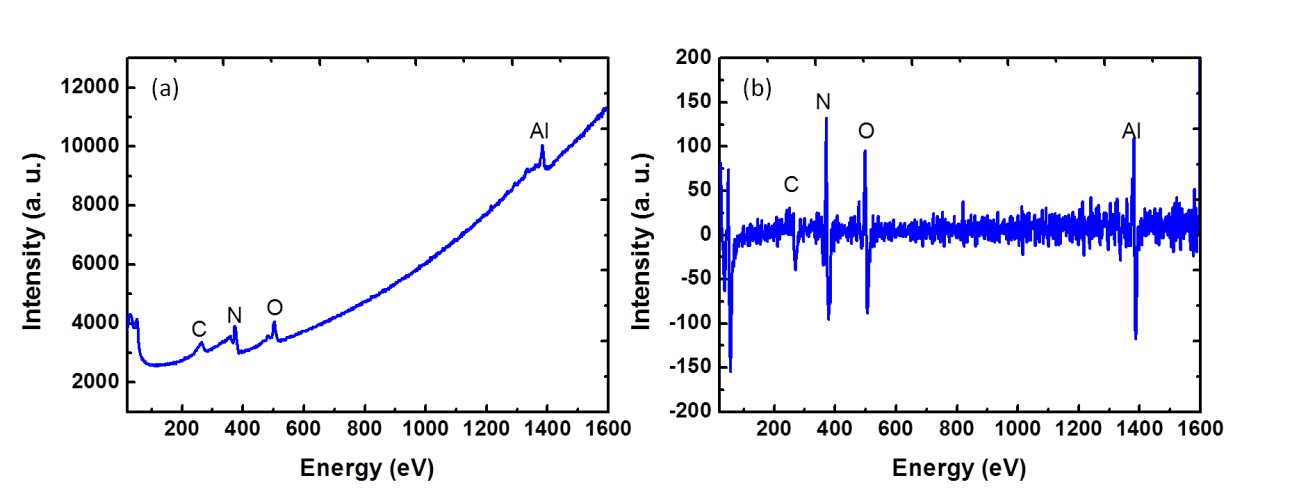


**Figure S5** (a) Direct and (b) differential Auger survey spectrum of the ALA-treated AlN layer.
